# Investigating amplitude of low-frequency fluctuation and possible links with cognitive impairment in childhood and adolescence onset schizophrenia: a correlation study

**DOI:** 10.3389/fpsyt.2024.1288955

**Published:** 2024-02-15

**Authors:** Yinghui Liang, Rongrong Shao, Yanhong Xia, Yadi Li, Suqin Guo

**Affiliations:** Department of Psychiatry, Henan Mental Hospital, The Second Affiliated Hospital of Xinxiang Medical University, Xinxiang, China

**Keywords:** amplitude of low-frequency fluctuation, children and adolescence onset schizophrenia, cognitive function, schizophrenia, RS-fMRI

## Abstract

**Background:**

Cognitive impairment (CI) is a distinctive characteristic of schizophrenia, with evidence suggesting that childhood and adolescence onset schizophrenia (CAOS), representing severe but rare forms of schizophrenia, share continuity with adult-onset conditions. While relationships between altered brain function and CI have been identified in adults with schizophrenia, the extent of brain function abnormalities in CAOS remains largely unknown. In this study, we employed resting-state functional magnetic resonance imaging (rs-fMRI) to investigate functional alterations in brain areas among patients with CAOS. To assess CI across multiple cognitive domains, we utilized the Stroop Color and Word Tests (SCWT) and MATRICS Consensus Cognitive Battery (MCCB) tests. Our objective was to explore the associations between functional CI and the amplitude of low-frequency fluctuation (ALFF) levels in these patients.

**Methods:**

We enrolled 50 patients diagnosed with CAOS and 33 healthy controls (HCs) matched for sex and age. Cognitive functions were assessed using the MCCB and SCWT methods. Rs-fMRI data were acquired using gradient-echo echo-planar imaging sequences. Voxel-based ALFF group maps were compared through two-sample t-tests in SPM8. Subsequently, correlation analyses were conducted to identify associations between ALFF levels and cognitive scores.

**Results:**

In comparison to HCs, patients exhibited significantly increased ALFF levels in the right fusiform gyrus, frontal lobe, and caudate, as well as the left frontal lobe and caudate. Conversely, reduced ALFF levels were observed in the temporal and left medial frontal lobes. Significant differences were identified between HCs and patients in terms of total cognitive scores, ALFF levels, and domain scores. All test scores were decreased, except for TMA. Correlation analyses between ALFF levels and cognitive functions in patients with CAOS differed from those in HCs. Pearson correlation analyses revealed positive associations between Brief Visuospatial Memory Test - Revised (BVMT-R) scores and ALFF levels in the left medial frontal gyrus. Digital Span Test (DST) scores were negatively correlated with ALFF levels in the right caudate, and Maze Test values were negatively correlated with levels in the left caudate. However, Pearson correlation analyses in HCs indicated that color and Hopkins Verbal Learning Test (HVLT-R) scores positively correlated with ALFF levels in the left frontal lobe, while color-word and symbol coding scores negatively correlated with levels in the right caudate.

**Conclusions:**

Altered ALFF levels in the brain may be linked to cognitive impairment (CI) in patients with CAOS. We highlighted the pathophysiology of schizophrenia and provide imaging evidence that could potentially aid in the diagnosis of CAOS.

## Introduction

Schizophrenia, as a mental disorder, still lacks a well-characterized etiology. Research has defined it as a neurodevelopmental disorder with a complex genetic basis (1, 2). Individuals with schizophrenia often exhibit cognitive dysfunction and negative symptoms that are frequently resistant to therapeutic interventions (3). Extensive studies have explored the relationships between brain structures and cognitive functions in schizophrenia, with cognitive remediation investigations focusing on both functional and connectivity changes (4, 5). Previous research has identified significant associations between overall brain structures and eight MATRICS-inspired cognitive domains, suggesting a potential functional network architecture underlying brain structure-cognition relationships (6). Other studies have provided comprehensive evidence of functional, structural, and neurochemical changes in the brain, particularly in subcortical regions and the association cortex (7). Social cognition, a significant research area in understanding cognitive impairment (CI) in schizophrenia, has linked changes in social cognition to structural and functional disturbances in areas known as the social brain (8). Cognitive remediation efforts have shown the ability to protect gray matter volume (GMV) in specific medial temporal lobes, such as the para-hippocampus, amygdala, and hippocampus, as well as thalamus regions. Meanwhile, functional alterations primarily impact the dorsolateral prefrontal lobe and insular cortex, both associated with improved cognitive ability (9). Notably, improvements in cognitive remediation have been observed in thalamic and prefrontal regions (10). Critically, neuroimaging facilitates the examination of affected brain areas linked with CI, and thus seeks to identify brain function alterations.

Childhood and adolescence onset schizophrenia (CAOS) represents a challenging psychiatric condition that is resistant to treatment, characterized by its chronic and severe nature, often surpassing the severity seen in adult-onset cases. Onset before adolescence is uncommon, but early age onset can predict worse prognosis (11). Unfortunately, there is a significant gap in the availability of safe and effective antipsychotics designed specifically for children and adolescents, making comprehensive research and development crucial for addressing CAOS. The rarity of CAOS is evident in a record-linkage study of hospital admissions in the UK. However, it is noteworthy that incidence rates of schizophrenia and non-affective psychoses have substantially increased among adolescents (12). Schizophrenia is widely regarded as a reflection of abnormal brain development, and its manifestation is particularly severe in patients with CAOS. Previous research on CAOS has suggested that developmental events may contribute to the onset of the condition (13). In comparative investigations focusing on CAOS, it has been observed that the severity of negative symptoms and the age of onset serve as pertinent predictors of the disease (14).

Previous advancements in neuroimaging have shed light on neural and functional abnormalities in early-stage schizophrenia, emphasizing the understanding of disease development in the maturing brain (15). In recent years, many neuroimaging-related schizophrenia investigations have reported that abnormal brain structures are related to pathophysiological actions underpinning schizophrenia (16, 17). In the present study, significant alterations in ALFF levels were recorded in the spontaneous oscillation of functional resting-state magnetic resonance images (rs-fMRI) (18, 19), and we observed that ALFF was a potential, biologically significant parameter for regional brain function assessment (20). Measuring ALFF levels through blood oxygenation-dependent signals has provided valuable insights into regional neural functions, showing correlations with local field potential activity (21). Importantly, few investigations have focused on CAOS in terms of both brain structure and function. Therefore, this investigation aims to contribute new insights into the pathophysiology of CAOS and scrutinize the diagnostic criteria for this complex disorder.

Cognitive dysfunction is a prominent feature in the majority of schizophrenia cases and is closely associated with the degree of social function prognosis in affected individuals. Patients commonly exhibit deficits in working memory, processing speed, attention, visual and verbal learning, as well as substantial impairments in reasoning, planning, problem-solving, and abstract thinking (22). In adults with schizophrenia, evidence suggests that mild cognitive impairment (CI) in those who later develop schizophrenia or related disorders may manifest in early childhood, potentially increasing the risk of schizophrenia in adulthood (23, 24). Compared to adult-onset schizophrenia, CAOS patients often experience more pronounced neurocognitive deficits, heightened disability levels, diminished socio-occupational functioning, and increased levels of self-stigma (25). A previous schizophrenia investigation reported that patients had poorer cognitive and social functioning during childhood and adolescence as well as present-state social cognition and cognitive functioning scores when compared to healthy controls (HCs) (26). While aberrant ALFF levels in brain areas and cognitive dysfunction are frequently reported in schizophrenia, the connections between ALFF and CI remain inadequately explored in patients with CAOS.

## Materials and methods

### Participants

Between October 2013 and March 2018, we recruited a total of 80 right-handed subjects after a thorough screening process, comprising 50 patients diagnosed with CAOS and 33 HCs. Ethical approval was obtained, and written informed consent was provided by all participants. CAOS diagnoses were determined using the Structured Clinical Interview for DSM-IV (SCID-IV), involving diagnostic discussions among at least two experienced clinicians. Patients with CAOS were aged between 8-18 years old, had a disease duration of less than 1 year, and were treatment-naïve to antipsychotic therapy. HCs from a local school underwent Structured Clinical Interview non-patient edition (SCID-NP) screening to ensure the absence of psychiatric and neurological illnesses in themselves and their immediate relatives. Clinical characteristics and demographics are presented in **Table 1**. Exclusion criteria included: (a) Intellectual Disability, (b) organic brain disorder, and (c) physical illness, including brain tumor, hepatitis or epilepsy as indicated by medical records. Brain MR ALFF BOLD data were assessed by experienced clinical personnel, and no gross abnormalities were identified.

**Table 1:** Participant clinical characteristics and demographics.

Characteristics	CAOS (n=50)	HCs (n=33)	*t*	χ^2^	*P*
Age (years)	14.22 ± 2.48	14.33 ± 2.70	-0.20	—	0.84
Sex, F:M	23:27	15:18	—	0.002	0.96
Education level (years)	8.22 ± 2.48	8.33 ± 2.70	-0.20	—	0.84
Illness duration (months)	5.26 ± 3.40	—	—	—	—

F, female; M, male; CAOS, childhood and adolescence onset schizophrenia; HCs, healthy controls.

“-” represents a value that does not exist.

### Data gathering

Functional and structural MRI scans were conducted using a 3T MR scanner (TIM Trio, Siemens, Erlangen, Germany) in a single session. For the generation of three-dimensional (3D) head images, rapid acquisition gradient echo sequences were employed with the following parameters: slice thickness of 1 mm, field of view (FOV) of 56 × 256 mm², repetition time (TR)/echo time (TE) of 530 ms/2.43 ms, and a flip angle of 7°. BOLD signal-sensitive MRIs were generated using gradient echo-planar imaging (EPI) sequences (TR/TE of 2000 ms/30 ms and a flip angle of 90°).

### Computing ALFF and preprocessing data

From each functional time series, we eliminated the first five volumes due to initial MRI signal instability and participant adaption to study conditions. Throughout rs-fMRI scans, head translation movement data of > 2 mm or > 2° rotation were removed. We slice-time-corrected EPI images, realigned them to initial first series images, and spatially normalized data to the Montreal Neurological Institute (MNI) EPI format, with voxels resampled to 3 × 3 × 3 mm^3^. We used the REST toolbox to perform T1-weighting, fMRI preprocessing, and ALFF image computation (26). After band-pass filtering (0.01–0.08 Hz)^10^ and linear trend removal, we converted time series’ to frequency domains using fast Fourier transformations (FFTs) (FFT length; shortest and taper percent; 0) to generate power spectra. As frequency power was proportional to component frequency amplitude squared, we square-rooted and averaged FFT power spectra across 0.01-0.08 Hz at each voxel to generate ALFF levels. For standardization, voxel ALFF levels were divided by global mean ALFF levels.

### Statistical analyses

The data were expressed as mean ± SD. Group comparisons were conducted using chi-squared tests and ANOVA in SPSS (version 17.0, IBM Corp, Armonk, NY, USA). Statistical significance was set at p<0.05.

## Results

### Demographic information

For sex (*P*=0.96), age (*P*=0.84), educational levels (*P*=0.84), handedness or head motion, no significant differences were observed between groups (**Table 1**). Participant movements were < 2 mm and < 2° for rotation.

### ALFF differences between groups

**Figure 1:**
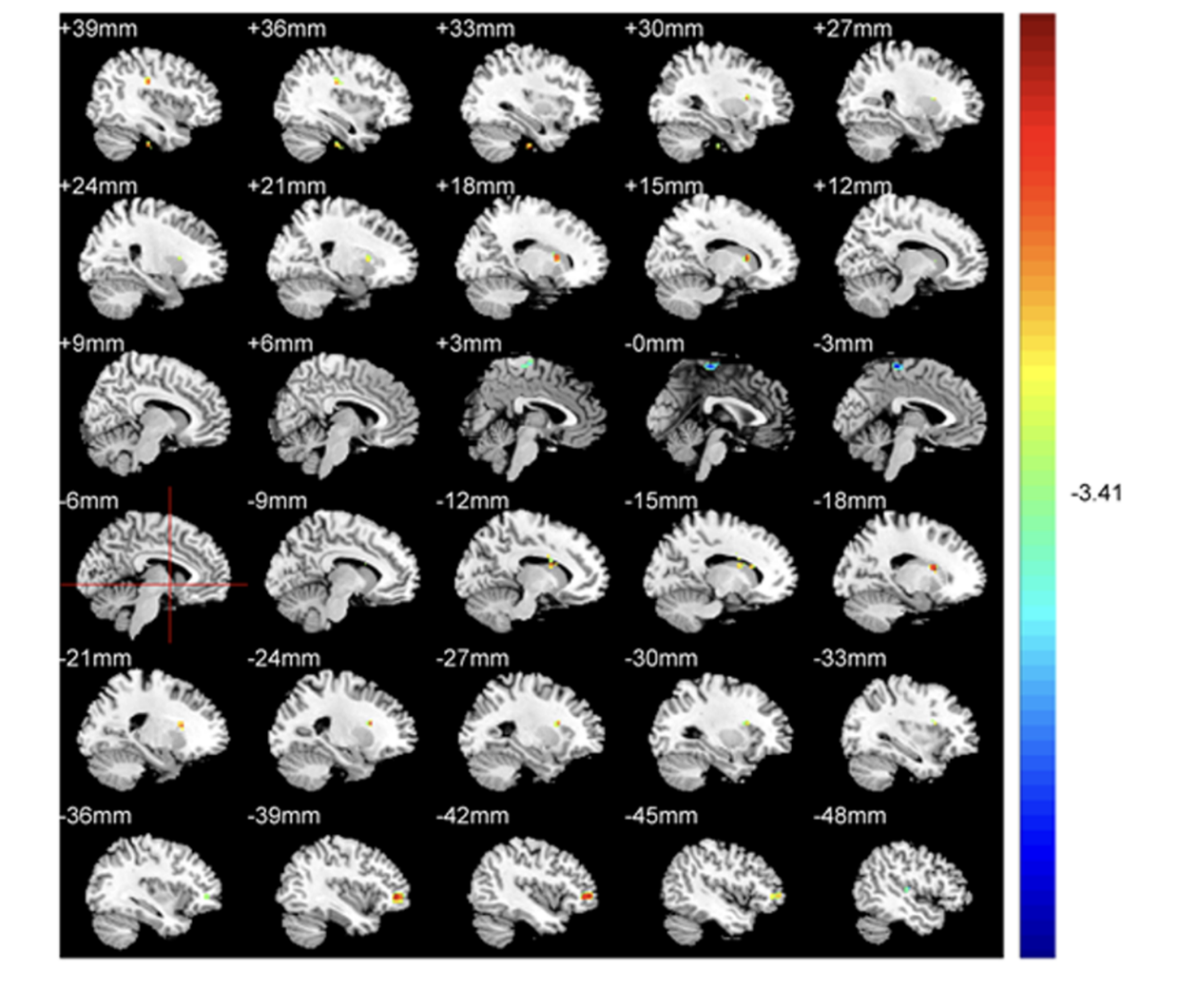
ALFF comparisons between patients with CAOS and HCs (*P*<0.05, AlphaSim corrected). Elevated ALFF levels in patients with COAS are red, while blue indicates reduced levels. When compared with HCs, patients recorded significantly elevated ALFF levels in the left frontal lobe and caudate, and right fusiform gyrus, frontal lobe, and caudate, while decreased levels were recorded in temporal and left medial frontal lobes.

We identified ALFF levels in the typical 0.01-0.08 Hz frequency band. In comparison to HCs, patients recorded significantly elevated ALFF levels in the left frontal lobe and caudate, as well as the right fusiform gyrus, caudate, and frontal lobe. Conversely, patients had significantly reduced ALFF levels in temporal and left medial frontal lobes (**Table 2**; **Figure 1**). Red areas indicated elevated ALFF levels in patients with respect to HCs, and blue areas showed lower levels in patients compared to HCs.

**Table 2:** ALFF (0.01-0.08 Hz) differences in HCs and patient brain areas.

Brain region	Cluster size	Peak coordinates (MNI)			T-values
		x	y	z	
L frontal lobe	42	-39	45	0	4.5848
L caudate	44	-18	15	15	4.8055
R fusiform gyrus	26	33	-18	-42	4.6110
R frontal lobe	17	39	-24	33	4.1542
R caudate	29	15	15	12	4.5811
L medial frontal	29	-3	-30	69	-4.6610
L temporal lobe	32	-51	-24	0	-4.9389

ALFF, amplitude of low frequency fluctuations; MNI, Montreal Neurological Institute; x, y, z, coordinates of peak locations in the MNI space.

### Cognitive outcomes

In comparison to HCs, patients with CAOS displayed significantly impaired total scores which were impaired across all domains. All test scores were reduced with the exception of TMA (*P*<0.001) (**Table 3**).

**Table 3:** Comparing cognitive functions in HCs and patients.

MCCB/SCWT	Patient group	Control group	*t*	*P*
TMA	63.74 ± 36.58	38.73 ± 13.35	4.41	<0.001
SC	37.64 ± 12.93	56.85 ± 9.57	-7.31	<0.001
HVLT-R	19.40 ± 6.07	26.85 ± 3.92	-6.79	<0.001
BVMT-R	18.60 ± 8.14	30.58 ± 2.76	-9.60	<0.001
FCF	14.32 ± 3.90	19.85 ± 4.12	-6.18	<0.001
DST	43.98 ± 14.52	78.03 ± 18.08	-9.48	<0.001
GML	11.32 ± 5.31	19.82 ± 4.25	-8.07	<0.001
word reading	63.72 ± 16.04	92.00 ± 16.39	-7.80	<0.001
color naming	42.24 ± 11.75	64.03 ± 13.95	-7.67	<0.001
named color-word	24.86 ± 8.77	35.42 ± 8.03	-5.55	<0.001

MCCB, MATRICS Consensus Cognitive Battery; SCWT, Stroop Color and Word Test; TMA, Trail Making Test: Part A; SC, Symbol coding; HVLT-R, Hopkins Verbal Learning Test-Revised; BVMT-R, Brief Visuospatial Memory Test-Revised; FCF, Fluency Category Fluency: Animal Naming; DST, Digital Span Test; GML, Groton Maze Learning; SCWT, Stroop Test conditions (word reading, color naming, named color-word).

### Correlations between cognitive function and ALFF

**Figure 2:**
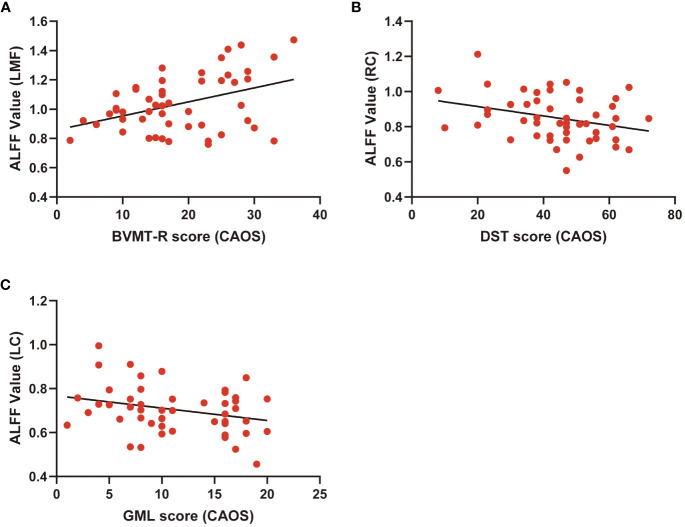
Scatter diagram showing relevant LMF trends in ALFF cluster levels, BVMT-R scores **(A)**, cluster RC of ALFF levels and DST scores **(B)**, LC of ALFF cluster levels, and GML scores **(C)**.

Correlations between ALFF levels and cognitive functions were examined in patients. Pearson correlation analyses indicated that BVMT-R scores positively correlated with ALFF levels in the left medial frontal gyrus (r=0.41, *P*<0.01). Digital Span Test (DST) scores negatively correlated with ALFF levels in the right caudate (r=-0.30, *P*<0.05) and Groton Maze Learning (GML) scores negatively correlated with ALFF levels in the left caudate (r=-0.28, *P*<0.05). Multiple correlation coefficient analyses were statistically significant (**Table 4**; **Figure 2**).

**Table 4:** ALFF and cognitive function correlations in patients.

	RFG	LFL	LTL	RC	LC	RFL	LMF
TMA	-0.02	0.04	0.03	0.04	0.08	0.13	0.02
SC	-0.06	0.10	-0.15	-0.07	-0.10	-0.13	0.03
HVLT-R	0.20	-0.10	-0.13	-0.08	-0.08	0.00	0.14
BVMT-R	-0.06	-0.06	-0.08	-0.14	-0.05	-0.10	0.41^**^
FCF	0.04	-0.04	0.01	-0.15	-0.03	0.12	-0.04
DST	0.00	0.02	-0.05	-0.30^*^	-0.22	0.05	0.05
GML	-0.01	-0.15	0.00	-0.16	-0.28^*^	0.00	0.25
word reading	0.14	0.17	-0.08	0.05	0.01	0.15	0.05
color naming	0.08	0.21	-0.07	-0.03	-0.05	-0.22	0.26
named color-word	0.08	0.06	-0.11	-0.14	-0.14	-0.03	0.12

*P<0.05, ** P<0.01; RFG, R fusiform gyrus; LFL, L frontal lobe; LTL, L temporal lobe; RC, R caudate; LC, L caudate; RFL, R frontal lobe; LMF, L medial frontal; TMA, Trail Making Test: Part A; SC, Symbol coding; HVLT-R, Hopkins Verbal Learning Test-Revised; BVMT-R, Brief Visuospatial Memory Test-Revised; FCF, Fluency Category Fluency: Animal Naming; DST, Digital Span Test; GML, Groton Maze Learning; SCWT, Stroop Test conditions (word reading, color naming, named color-word).

Bold values means the correlation between the scores of cognitive function test items and the ALFF values of brain regions with significant differences. * denotes P < 0.05.

**Figure 3:**
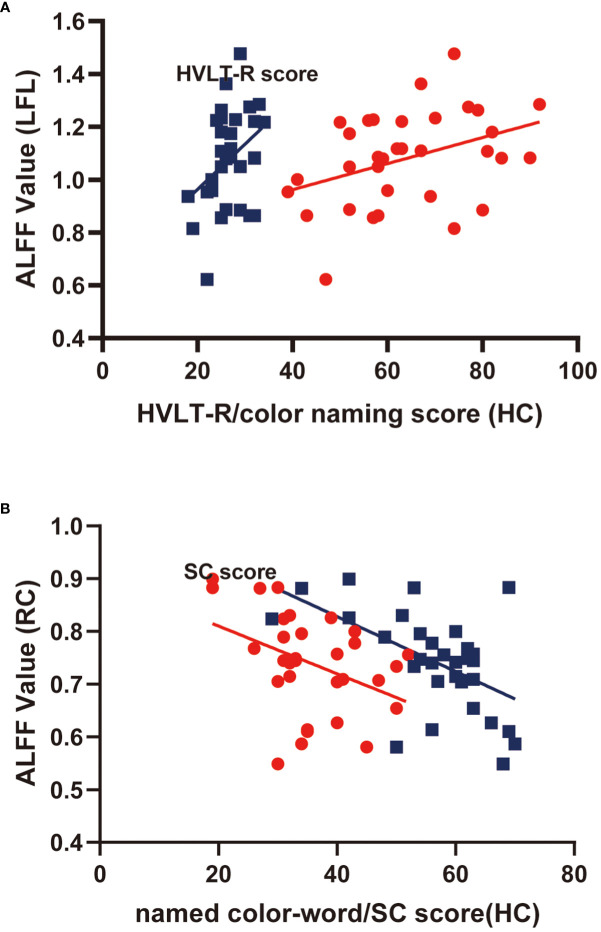
Scatter diagram showing relevant LFL trends in ALFF cluster levels, HVLT-R scores, color-naming scores **(A)**; RC of ALFF cluster levels, named color-word scores, and SC scores **(B)**.

Correlations in HCs were also examined; Pearson correlation analyses indicated that color-naming and HVLT-R scores positively correlated with ALFF levels in the left frontal lobe (r=0.37, 0.38, *P*<0.05). Conversely, named color-word scores and symbol coding negatively correlated with ALFF levels in the right caudate (r=-5.48, -0.40, *P*<0.05). The findings from multiple correlation coefficient analyses were also statistically significant (**Table 5**; **Figure 3**).

**Table 5:** ALFF and cognitive function correlations in HCs.

	RFG	LFL	LTL	RC	LC	RFL	LMF
TMA	0.26	-0.11	0.15	0.07	0.23	-0.05	0.31
SC	-0.21	0.10	0.01	-0.55*	-0.24	0.04	0.30
HVLT-R	-0.16	0.37*	0.23	-0.17	-0.21	-0.06	0.01
BVMT-R	0.19	0.08	0.12	0.26	0.21	0.18	-0.07
FCF	-0.01	0.01	0.08	0.02	-0.07	-0.21	0.26
DST	-0.10	0.07	-0.08	0.10	0.00	-0.05	-0.04
GML	-0.04	-0.11	0.30	-0.09	-0.03	-0.20	0.21
word reading	-0.02	0.30	-0.02	-0.18	-0.06	-0.17	0.04
color naming	0.13	0.38*	0.05	-0.24	-0.07	-0.02	0.26
named color-word	0.21	0.23	0.11	-0.40*	-0.22	0.01	0.12

*P<0.05; RFG, R fusiform gyrus; LFL, L frontal lobe; LTL, L temporal lobe; RC, R caudate; LC, L caudate; RFL, R frontal lobe; LMF, L medial frontal; TMA, Trail Making Test: Part A; SC, Symbol coding; HVLT-R, Hopkins Verbal Learning Test-Revised; BVMT-R, Brief Visuospatial Memory Test-Revised; FCF, Fluency Category Fluency: Animal Naming; DST, Digital Span Test; GML, Groton Maze Learning; SCWT, Stroop Test conditions (word reading, color naming, named color-word).

Bold values means the correlation between the scores of cognitive function test items and the ALFF values of brain regions with significant differences. * denotes P < 0.05.

## Discussion

In previous years, researchers have used the ALFF method to examiners local brain activity in adult patients with schizophrenia and identified links between CI and altered brain functions (18, 21). In our investigations, ALFF differences and CI levels were identified between our groups. When compared with HCs, patients displayed elevated ALFF levels in the left frontal lobe and caudate, as well as the right fusiform gyrus, frontal lobe, and caudate, Conversely, decreased levels were noted in the temporal and left medial frontal lobes. In patients with CAOS, we identified positive correlations between BVMT-R scores and ALFF levels in the left medial frontal gyrus, but negative correlations were present between DST scores and ALFF levels in the right caudate, and GML scores and ALFF levels in the left caudate. We also examined correlations in HCs; color-naming and HVLT-R scores positively correlated with ALFF levels in the left frontal lobe, whereas color-word scores and symbol coding negatively correlated with ALFF levels in the right caudate. Importantly, our study marked the first exploration of relationships between cognitive function and ALFF levels in patients with CAOS.

ALFF levels serve as indicators of spontaneous and intrinsic neuronal activity, commonly used to highlight abnormalities in conditions like schizophrenia (19). In our study, when compared with HCs, patients with CAOS displayed aberrant ALFF levels in numerous brain areas. A previous meta-analysis reported that spontaneous brain activity alterations in drug-naïve first-episode schizophrenia predominantly included the frontal lobe, putamen, and cerebellum (27). Our findings aligned with this, revealing significantly elevated ALFF levels in the frontal lobe, consistent with previous studies. Our rs-fMRI data further indicated increased elevated ALFF levels in the bilateral caudate, consistent with patients with schizophrenia (28, 29). Also, previous investigations described elevated ALFF levels in the right hippocampus and left caudate (30, 31). In comparison with HCs, patients with schizophrenia had lowered ALFF levels in the left angular gyrus and fusiform gyrus (32). Our study also identified elevated ALFF levels in the right fusiform gyrus and reduced levels in the left temporal lobe, which partly agreed with Alonso-Solís et al. (33), although they also reported elevated ALFF levels in the temporal pole, in contrast with our observations. A previous rs-fMRI investigation identified elevated ALFF levels in the medial frontal gyrus, and these data were confirmed in a first-episode schizophrenic patient investigation (34, 35). However, in our investigation, we identified reduced ALFF levels in the left medial frontal gyrus. These heterogeneous observations are unfortunate and may be reflected by medication effects, age, or illness duration. To limit such factors, we selected first-episode children and adolescents. Importantly, our findings suggested that abnormal ALFF levels in patients with COAS could distinguish these patients from healthy children and adolescents.

Cognitive Impairment (CI) is a fundamental trait in schizophrenia, characterized by consistent deficits across various cognitive domains (23, 25). Numerous reviews have highlighted that individuals with schizophrenia exhibit heightened CI compared to both HCs and individuals with affective disorders, particularly in memory and processing speed domains (36). A previous review also reported that presence of mild CI in early childhood among individuals who later develop schizophrenia, with a notable increase in CI during adolescence, the prodrome, and the first psychotic episodes (24). In our study, the impaired cognitive functions observed in patients with COAS concurred with previous investigations showing CI in all domains in schizophrenia patients. The MATRICS Consensus Cognitive Battery (MCCB) was used to assess the following cognitive domains: 1) visual learning 2) vigilance/attention, 3) working memory, 4) social cognition, 5) processing speeds, 6) problem solving/reasoning, and 7) verbal learning (37). Stroop word-reading reflected visual-search speed, Stroop color-naming indicated working memory and visual-search speed, and Stroop color-word referred to working memory, conflict monitoring, and visual-search speed (38). When MCCB and Stroop Color and Word Test (SCWT) methods were used to measure cognitive functions, all test scores were lower except for TMA. A recent investigation indicated that cognitive profiles in childhood and adolescence could be used to differentiate between psychiatric disease spectra (39). Our data further suggested that CI could serve as a distinguishing factor for patients with COAS compared to healthy children and adolescents, and the degree of impairment may contribute to prognosis considerations.

Individuals with schizophrenia commonly exhibit cognitive domain deficits, yet the neurobiological underpinnings of these impairments remain largely unknown (3). Neuroimaging studies have highlighted the neurobiological foundation of cognitive function in schizophrenia, and facilitated exploration of affected brain areas associated with such CI’s. Recently, it was reported that neurocognition was associated with brain structures which was characterized by higher immediate recall scores which were linked to elevated GMV in the left temporal pole, elevated verbal fluency scores associated with elevated GMV in the left temporal pole, middle temporal gyrus, and elevated Stroop-word scores linked to raised GMV in the right middle frontal gyrus (40). Previous findings also reported that executive function impairments were linked to prefrontal cortex thickness and volume, cognitive control impairments were correlated with elevated anterior cingulate cortex activation, and episodic memory impairments were related to hippocampal reduction (10). From fMRI analyses, BVMT-R scores in patients were positively linked to ALFF levels in the left medial frontal gyrus. Also, prefrontal cortical deficits occurred during schizophrenia and reflected neural working-memory pathway deficits, consistent with elements of our investigation (41, 42). Moreover, HVLT-R and color-naming scores in HCs were positively linked with ALFF levels in the left frontal lobe. Importantly, these aforementioned research studies exemplified the relationship between the frontal lobe and cognition.

While early investigations in schizophrenia indicated that ALFF levels in the left precentral gyrus negatively correlated with several MCCB cognitive domains, and opposite results in the left cortex/precuneus (31). Compared to HCs, patients exhibited decreased ALFF in the bilateral postcentral gyri and paracentral lobule, and these reductions were negatively correlated with the symbol coding sub-tests of MCCB (28). Additionally, in patients, DST scores negatively correlated with ALFF levels in the right caudate, while GML scores negatively correlated with levels in the left caudate. However, overall, research findings in this area are inconsistent across studies, and our understanding of the relationships between functional CI and ALFF remains limited.

Our study presented several strengths. Firstly, links between ALFF levels and CI in patients with COAS have never been studied before. Secondly, our study circumvented confounding factors, such as medication effects, illness chronicity, and educational background, in affected individuals. Finally, we examined relationships between brain function and CI in patients with COAS to better understand the pathophysiology underlying schizophrenia and improve prognostic social functioning. Nevertheless, our investigation had certain limitations. Firstly, COAS is a rare mental disorder, therefore subject recruitment was limited, and sample size was low. Secondly, we were unable to eliminate all physiological noise effects. Thirdly, some participants may not have shut their eyes during resting sessions. Lastly, while we limited head movements as much as possible, a previous investigation indicated that minimal head motion affected ALFF measurements during rs-fMRI (43). Therefore our future aim is to expand sample sizes and comprehensively address these limitations.

## Conclusions

CI is prominent in patients with CAOS who presented with ALFF alterations in several brain areas during our investigations - these changes may be linked to CI in these patients. Critically, our investigation provides new information on the pathophysiology of schizophrenia and provides improved diagnostic and predictive insights for patients with CAOS.

## Data availability statement

The raw data supporting the conclusions of this article will be made available by the authors, without undue reservation.

## Ethics statement

The studies involving humans were approved by The Second Affiliated Hospital of Xinxiang Medical University. The studies were conducted in accordance with the local legislation and institutional requirements. The participants provided their written informed consent to participate in this study. Written informed consent was obtained from the minor(s)’ legal guardian/next of kin for the publication of any potentially identifiable images or data included in this article.

## Author contributions

YiL: Data curation, Formal Analysis, Writing – original draft. RS: Formal Analysis, Software, Writing – original draft. YX: Methodology, Project administration, Visualization, Writing – original draft. YaL: Data curation, Formal Analysis, Writing – original draft. SG: Writing – review & editing.
